# Antidiabetic Properties of the Tropical Tree *Schinus molle* L. (pirul): A Comprehensive Review

**DOI:** 10.3390/ph18111661

**Published:** 2025-11-02

**Authors:** Rosa María Fonseca, Maira Huerta-Reyes

**Affiliations:** 1 Laboratorio de Plantas Vasculares, Departamento de Biología Comparada, Facultad de Ciencias, Universidad Nacional Autónoma de México, Ciudad Universitaria, Coyoacán, Ciudad de México 04510, Mexico; 2 Unidad de Investigación Médica en Enfermedades Nefrológicas, Hospital de Especialidades “Dr. Bernardo Sepúlveda Gutiérrez”, Centro Médico Nacional Siglo XXI, Instituto Mexicano del Seguro Social, Cuauhtémoc, Ciudad de México 06720, Mexico

**Keywords:** diabetes mellitus, *Schinus molle*, pirul, antioxidant, anti-inflammatory, antidiabetic, α-glucosidase, α-amylase

## Abstract

The need for new medications to treat diabetes mellitus (DM) is a global health concern due to the cost and impact on patients and their families, health systems, and society. Recent approaches in drug development have focused on multitarget therapy for DM, considering its multifactorial and complex pathophysiology. The present work contributes to the review of the plant species *Schinus molle* L. (pirul), a tropical tree native to South America but now widespread worldwide, which has demonstrated anticancer, analgesic, antibacterial, and insecticidal properties. According to traditional uses, pirul has been employed as a food condiment, in the preparation of beverages and chewing gums, and in the treatment of DM. The antidiabetic effects of pirul appear to act through several mechanisms involved in DM. The methanolic extract of *S. molle* fruits collected in Tunisia exhibited a dose-dependent inhibition on both α-amylase and α-glucosidase enzymes (77.49% and 86.45%, respectively). A dose-dependent anti-inflammatory effect was also observed at 1, 2, 3, 4, and 5 h, in the carrageenan-induced rats’ paw edema model. Furthermore, in both the H_2_O_2_ and the superoxide radical assays, the pirul extract demonstrated moderate antioxidant activity (IC_50_ = 0.22 mg/mL). Isomasticadienonic acid and Masazino-flavanone, the major components of active fractions and extracts of *S. molle* represent promising antidiabetic agents. Although pirul appears to be safe in in vivo acute and subchronic administrations, toxicological studies and clinical trials in individuals with DM are still pending.

## 1. Introduction

DM is a chronic disease that can be considered one of the most common and fastest-growing worldwide. According to the World Health Organization (WHO), its prevalence has been increasing alarmingly, as the number of persons living with DM has quadrupled over a 30-year period, rising from 200 million in 1990 to 830 million in 2022 [1]. The absolute global economic burden will increase from USD 1.3 trillion in 2015 to USD 2.2 trillion in the baseline scenario and to USD 2.1 trillion in the target scenarios for 2030. This represents an increased cost as a percentage of global GDP from 1.8% in 2015 to a maximum of 2.2% for the year 2030. Therefore, the economic burden of DM is considerable for patients and their families, for health systems, and for societies as a whole. The costs associated with diabetes are substantial and include not only medical expenses but also indirect costs related to disability, premature death, and loss of productivity [2]. As a consequence, the management of DM requires immediate actions and alternatives that positively impact prevention and pharmacological treatments for these to be more effective and less expensive. Recent research considers a comprehensive approach in the treatment of DM, taking into account its multifactorial and complex pathophysiology. Thus, plant extracts and their metabolites exhibited numerous physiological activities that could be involved in different pathways of the disease, offering a potential alternative therapy for DM [3]; among these activities are potent antioxidants, anti-inflammatories, hypoglycemics, hepatoprotectors, cardiovascular protectors, and nephroprotectors [4,5,6,7]. Therefore, the need to study plants that can exhibit possible multitarget functions useful in the treatment of DM appears essential. In this review, we present *S. molle*, a tropical tree native to South America but currently broadly distributed worldwide. Although there are some reports in traditional medicine on its use for the treatment of DM [8], its experimental antidiabetic properties have scarcely been considered. Thus, this review aims to be a contribution to the area of research of natural products as potential drugs that could be applicable in the clinical treatment of DM in the near future.

## 2. Overview of *S. molle*

### 2.1. Botanical Description and Geographical Distribution

*S. molle* is a tree native to South America, Peru, northern Chile, and from southern Brazil to northeastern Argentina that belongs to the botanical family Anacardiaceae. It has been introduced in several regions of North America, Asia, Africa, and Europe, and the genus *Schinus* consists of about 40 species, all endemic to South America [9]. *S. molle* is known by different common names; in Mexico, it is known as piru, pirúl or pirul, tree of Peru, pirwi, tsactumi, tzactumi, tzantuni, xasa, xaza, peloncuáhuitl, yaga-cica, and yaga-lache [10]. In Brazil, it is called aroeira, aroeira-salsa, periquita, anacauíta or anacahuita, and molho [11]. In some other regions, it is also known as aguaribay, aguaribay pepper, aguacibay, molle, Bolivian molle, pimentero, Peruvian pepper, paprika, wood pepper, poivrier d’america, quebracho Colorado, and terebinto [12].

**Figure 1:**
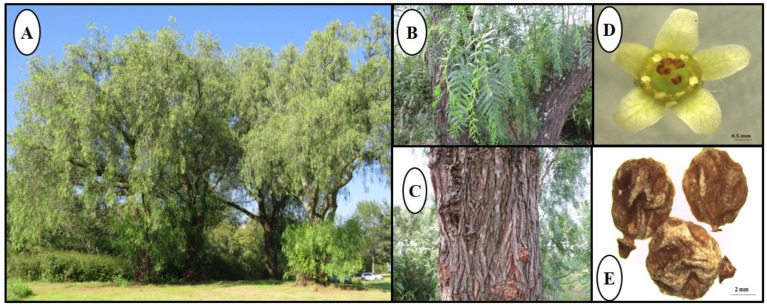
Detail of the tropical tree *S. molle*: (**A**) habit; (**B**) leaves; (**C**) cortex; (**D**) flower, and (**E**) dried seeds. Photos: Rosa María Fonseca.

*S. molle* is a tree that can reach up to 10 m in height and is recognized by its thin, hanging branches, with leaves 15–30 cm in length divided into 17–35 lanceolate leaflets, each 1.5–5 cm in length, 2–10 mm in width, with the entire margin or sometimes serrulate, without trichomes, or very rarely, with few trichomes, flowers in clusters (September–March), branched, hanging, up to 30 cm in length, and the fruits more or less spherical, 5–7 mm in diameter [12,13] (Figure 1).

Barkley observed, in the 1950s [12], that the pirul grew in many temperate regions of the world because it was distributed by the Spanish colonialists since the 16th century, as an ornamental plant and producer of fruits with a flavor similar to that of pepper. The pirul is now part of the landscape of many places in the world, and, in some countries like Mexico, due to its great abundance, it could appear to be native. Among the multiple applications of this species, its reforestation and ecological restoration exhibited notable results due to its resistance to various environmental factors [14].

### 2.2. Uses and Biological Properties of Pirul

From pre-Columbian times, in South America, a drink called *chicha* has been prepared with the fruits of *S. molle* containing beneficial probiotics [15,16]. The dry fruits have been used as a condiment in some countries to adulterate black pepper due to their similar flavor, although their use is becoming less common [17]. The resin has been used as a base for chewing gum in order to strengthen the gums. Dyes are also obtained from the leaf bark, stem, bark, and root [17,18].

The pirul is linked to various practices of the native cultures of Mexico, such as the Mixe, the Zapotec, and the Totonaca, which employed its branches and leaves in purification rituals denominated as “cleasings” (*limpias*), to remove “the bad air”, “the evil eye” and the “scare” (*espanto*); for this reason, in numerous states of the country, the branches and leaves of the pirul are sold in popular marketplaces [19]. In Inca culture, the resin of *S. molle* was employed for embalming corpses [20].

One of the most widespread traditional uses of the pirul is as a repellent. In rural communities in South America and Mexico, residents collect the fruits of the pirul to repel flies and mosquitoes in their homes, on their livestock, and on their crops [21]. The effectiveness of essential oils from pirul leaves and fruits has also been reported for the control of disease transmission vectors such as chikungunya and Zika [22,23], as well as in the control of pests such as mites in bee hives [24] and in goats [25]. The essential oil and hexanic extracts of *S. molle* showed potential as a repellent for the oriental cockroach (*Blata orientalis*) [26]. The insecticidal activity of the essential oil of the pirul by contact showed acceptable results in the exposure method of the adults of *Sitophilus oryzae*; thus, the fruits of *S. molle* are more active against this species of beetle than the essential oil of *S. terebinthifolius*. The majority of the compounds present in the essential oil of the leaves and fruits of *S. molle* are *β*-Pinene (10.36–5.44%), *γ*-Terpinene (12.01–8.15%), Limonene (22.94–18.49%), 10-epi-elemol (7.64–8.03%), *γ*-Eudesmol (5.17–4.09%), and Longifolene (7.67–8.48%). Also detected was the presence of the following compounds that are only present in the essential oils of the species *S. molle* and absent in the species *S. terebinthifolius*: γ-Muurolene, γ-Gurjunene, γ-Cadinene, 10-epi-elemol, Guaiol, and α-Acorenol [27]. The effectiveness of an ointment containing *S. molle* essential oil was observed in cattle with infected wounds [28]. Currently, the essential oils of the pirul are employed as a cosmetic ingredient with anti-pollution, anti-aging, and anti-wrinkle properties [29].

Given its traditional use against toothache, chipped teeth, and wound healing, an experimental study was carried out, where the result shows that the resin of *S. molle* demonstrated a cariostatic effect. On the other hand, the analgesic effect of the dichloromethane extract of the leaves of the pirul has been confirmed for relieving rheumatism and muscle pain [30].

Some evaluations concerning the cytotoxicity activities of the essential oils of *S. molle* were carried out on hepatocellular, colon, and breast carcinoma cell lines. Fruit oil showed the greatest potency against the colon and hepatocellular cell lines, while flower oil exhibited the most potent activity against breast-carcinoma cell lines. Therefore, the specific activities on every different cell line suggested the future investigation of *S. molle* in different types of cancer [31].

The wood of *S. molle* has an acceptable paper pulp quality index [32]. The study of wood chemistry proposes its possible use for the kraft pulp process and as an excellent candidate for paper pulp [33,34]. As fuel the wood of the pirul has been classified as adequate due to its calorific value, which is near that of the wood of some pines and oaks [34].

However, pirul has been considered as an invasive species [19,35] and as a harmful plant in Africa (Noxious Weed), in that it competes with native species [36]. Later studies on the ecological role of *S. molle* revealed its positive interaction with a number of native animal species, as well as the advantage of the pirul for its self-reproduction, without human intervention, which has been favored by its cultural importance [19].

## 3. Therapeutic Properties of Pirul as Related to DM

### 3.1. Inhibition of the Enzymes α-Glucosidase and α-Amylase

In recent decades, the decrease in the post-prandial glucose levels through the inhibition of the degradation of the oligo- and disaccharides has been considered as a therapeutic strategy in the treatment of DM. One of these strategies focused on the inhibition of the enzymes α-amylase and α-glucosidase, which play a key role in the digestion of starch and glycogen, and consequently it improves glycemic control, which is also observed in a reduced glycosylated hemoglobin level. Acarbose and miglitol are α-glucosidase inhibitors that are FDA-approved drugs in the treatment of DM that can be used alone or combined with other antidiabetic drugs [37,38]. Several plant extracts have been investigated as potential α-amylase and α-glucosidase inhibitors as therapeutic alternatives in order to mitigate the secondary effects of the already available drugs [39]. In this context, the methanolic extract of the fruits of *S. molle* exhibited a dose-dependent inhibition on both enzymes, that is, α-amylase and α-glucosidase. The extract exerted percentages of inhibition of 77.49 and 86.45 for α-amylase and for α-glucosidase at 0.4 mg/mL concentration, respectively. A thorough pharmacological profile requires testing against multiple targets and in different model systems. In the same study, this enzymatic inhibition of the extract of *S. molle* fruits was even higher than that of the control acarbose and even higher than other species belonging to the same genus, that is, *Schinus*, such as the *S. terebinthifolius* methanolic extract of the fruits (80.31%) for the case of the inhibition of α-glucosidase enzyme. The major compound identified in this methanolic extract was Masazino-flavanone (Table 1) [40]. In the same manner, the monosaccharide fraction of the fruits of *S. molle* presented a higher inhibition in the α-glucosidase enzyme when compared with the control ascorbic acid (IC_50_ = 0.17 mg/mL and IC_50_ = 0.25 mg/mL, respectively) and with the nearby species from the same genus, *S. terebinthifolius (*IC_50_ = 0.22 mg/mL). In the case of the inhibition on the α-amylase enzyme, again, the monosaccharide fraction of *S. molle* exerted a potent inhibition, higher than that of the control ascorbic acid (IC_50_ = 0.16 mg/mL and IC_50_ = 0.21 mg/mL, respectively) [41]. The monosaccharide composition is detailed in Table 1. In a different study, the aqueous extracts of *S. molle* exhibited a potent inhibition effect in an α-glucosidase assay (>80%), but, in contrast, the inhibition of this same extract on the α-amylase enzyme was low (<25%) [42]. With similar results, the study carried out by İlgün et al. [43] exhibited a potent inhibition on the enzyme α-glucosidase by the methanolic extracts of the leaves (94.74 ± 5.61%), raw fruit (99.11 ± 1.61%), and ripe fruit (98.64 ± 1.00%) of *S. molle*, even higher than the control acarbose (88.60 ± 0.64%). In contrast, the methanolic and aqueous extracts of the leaves, raw fruit, and ripe fruit of *S. molle* were inactive on the α-amylase enzyme assay. Additionally, the antidiabetic activity of the extracts was evaluated in the glucose-induced diabetes model of β-TC cells (pancreatic β cells) by measuring glucose and insulin levels of the β-TC cells. However, both the aqueous and the methanolic extracts of the leaves, raw fruits, and ripe fruits of *S. molle* did not show any activity on this in vitro experimental model [43].

### 3.2. Anti-Inflammatory Effect

Inflammation is the physiological response of the body to infections or tissue damage. Subclinical chronic inflammation is frequently present during the natural course of DM and is reflected in elevated levels of inflammatory biomarkers. Numerous investigations have confirmed the role of inflammation in both the onset and progression of diabetes [44,45]. Chronic inflammation has been recognized as a key contributor to DM and other metabolic disorders such as insulin resistance and obesity. Abnormal cytokine production, elevated acute-phase reactants and other mediators, as well as the activation of inflammatory signaling networks, are characteristic features of chronic inflammation. Among the cytokines considered central to inflammatory processes is Tumor Necrosis Factor-α (TNF-α), currently regarded as the primary link between obesity, diabetes, and chronic inflammation due to its overexpression in the adipose tissue of obese mice [46]. Other relevant cytokines include Interleukin-6 (IL-6) and Interleukin-1 beta (IL1β), which promote insulin resistance by interfering with insulin signaling in peripheral tissues. Clinical data have demonstrated that adipose tissue, liver, muscle, and pancreas are the principal sites of inflammation in DM [47]. Given that several plant-natural products possess potent anti-inflammatory properties acting through different mechanisms and that many of them are derived from edible species, anti-inflammatory activities are increasingly recognized as important therapeutic targets in DM. Consequently, the search for and the identification of new anti-inflammatory metabolites have intensified in recent years [48].

The in vivo anti-inflammatory properties of the methanolic extract of *S. molle* fruits were evaluated in the carrageenan-induced rat paw edema model. A dose-dependent anti-inflammatory effect was observed at 1, 2, 3, 4, and 5 h, with the strongest effect detected at 100 mg/kg [40]. For the monosaccharide fraction obtained from *S. molle*, maximal inhibition was observed at 5 h, reaching 64.66%, which was comparable to that of the control Indomethacin (70.64%) [41].

Another investigation on Sprague Dawley rats evaluated the in vivo anti-inflammatory properties of two fractions derived from the dichloromethane extract of *S. molle* seeds. Both fractions exhibited potent anti-inflammatory effects at 1 and 2 h; however, at 4 and 5 h, fraction 2 showed significantly anti-inflammatory activity when compared with the control Ibuprofen [49].

Ethanolic extracts of *S. molle* fruits and leaves from Syria were evaluated in in vitro (protein denaturation inhibition) and in vivo (carrageenan-induced edema) assays. At a concentration of 200 μg/mL, the ethanolic extract of the fruits showed maximal in vitro anti-inflammatory (75.3 ± 1.1%), which was more potent than the positive control (Diclofenac), even at a higher concentration of 300 μg/mL. The ethanolic extract of leaves also exhibited a dose-dependent anti-inflammatory effect, with maximal activity observed at 200 μg/mL (50.8 ± 7.6%), comparable to the control Diclofenac. In the in vivo assay, although the ethanolic extract of fruits reduced edema after 1 and 2 h, the highest anti-inflammatory activity was recorded at 4 h (73 ± 3.16%), which was moderate compared with the control Diclofenac (88 ± 0.04%) [50].

The anti-inflammatory activities of two triterpenoids (3-*epi*-Isomasticadienolalic acid and Isomasticadienonalic acid) and one biflavanone (Chamaejasmin) isolated from the fruits of *S. molle* (Table 1) were evaluated using two different assays: the first focused on acute inflammation (Phospholipase A_2_ [PLA_2_]-induced paw edema), and the second on chronic inflammation (repeated administration of 12-O-tetradecanoylphorbol-13-acetate [TPA]). The results showed that only the triterpene Isomasticadienonalic acid was active in the PLA_2_ model, exhibiting 66% at a dose of 30 mg/kg at 60 min. In contrast, in the chronic inflammation model, both the triterpenes and the biflavanone produced swelling reductions ranging from 48 to 26%, indicating that *S. molle* contains anti-inflammatory molecules acting through different metabolic pathways [51].

### 3.3. Antioxidant Effect

The relevance of oxidative processes in the pathogenesis of DM has been widely recognized in recent decades. An imbalance between oxidants and antioxidants can lead to early vascular dysfunction and trigger proinflammatory responses that are exacerbated during the progression of DM. This imbalance results from the disruption of equilibrium between vascular superoxide/H_2_O_2_ production and/or the decline of antioxidant defenses [52,53]. Antioxidant agents derived from plants act as free radical scavengers capable of controlling the harmful effects of these unstable species in the human body and, therefore, may be useful in the treatment of DM and its complications [54]. For this reason, the antioxidant activities of the methanolic extracts of *S. molle* fruits were evaluated by three complementary in vitro assays, namely, the scavenging ability of hydrogen peroxide (H_2_O_2_), the scavenging ability of the superoxide radical, and the ABTS assay. In the ABTS assay, the methanolic extract of *S. molle* showed a strong free radical-quenching effect, as it is more efficient than the control BHT (>80%). Conversely, in both the H_2_O_2_ and the superoxide radical assays, the methanolic extract demonstrated moderate activity (66.47% at a concentration of 0.3 mg/mL) and exhibited an IC_50_ = 0.22 mg/mL with respect to the control BHT, revealing a strong scavenging capacity with values of 87.45% and IC_50_ = 0.12 mg/mL, respectively [40].

In the DPPH assay, one of the most common methods to assess the antioxidant capacity in plant extracts, the monosaccharide fraction of *S. molle* fruits exhibited a dose-dependent effect with an IC_50_ value of 0.22 mg/mL. In the ABTS assay, this same fraction showed a similar dose-dependent effect with a value of IC_50_ = 0.28 mg/mL. In both cases, the antioxidant effects were considered moderate when compared to the control. In the inhibition of H_2_O_2_-induced erythrocyte oxidative hemolysis, the monosaccharide fraction exhibited dose-dependent antihemolytic activity with an IC_50_ = 0.23 mg/mL. Although the control demonstrated stronger antioxidant activity than the monosaccharide fraction of *S. molle* fruits, this erythrocyte oxidative hemolysis was still considered relevant when compared with other plant extracts [41]. Another study revealed that the aqueous extracts of *S. molle* exhibited low antioxidant activity in the DPPH assay (<50%), which is likely related to their low chlorogenic acid content (Table 1) [42].

Kim et al. [55] investigated the antioxidant effects of methanolic extracts of *S. molle* fruits from different countries, including Brazil, India, and Sri Lanka. In both the DPPH and ABTS assays, the strongest antioxidant activity was observed in samples from India (4081.92 ± 34.39 mg VCE/100 g and 2845.12 ± 3.91 mg VCE/100 g, respectively), while the lowest antioxidant capacity was detected in samples from Sri Lanka (2812.30 ± 10.81 mg VCE/100 g and 1956.96 ± 54.26 mg VCE/100 g, respectively). The work of Martins et al. [56] focused on the essential oils extracted from the leaves and fruits of *S. molle* (Table 1). In the DPPH assay, inhibition percentages were low (leaf, 4.8% and fruit, 5.5%) when compared with the control ascorbic acid (>14%). However, when these essential oils were evaluated using the β-Carotene bleaching method, which measures the inhibition of β-Carotene oxidation by peroxides generated during linoleic acid oxidation, the results showed remarkable antioxidant effects (leaf, 57% and fruits, 19%), far exceeding that of the ascorbic acid control (1%). Similarly, IC_50_ values calculated for leaf (0.8 mg/mL), fruits (4.2 mg/mL), and control (17.4 mg/mL) indicated that these essential oils are more effective antioxidants through lipid peroxidation inhibition than through direct radical scavenging. Another study evaluating essential oils from *S. molle* wood branches revealed potent antioxidant activity (90 ± 1.23%), even higher than that of the control catechin (84.13 ± 1.90%) [57].

A separate investigation on the antioxidant activities of methanolic extracts from *S. molle* seeds and leaves from Saudi Arabia (Table 1) using the DPPH assay, showed maximal scavenging activities of 78.32% and 75.46%, respectively, at 1000 μg/mL, values considered moderate, given that the ascorbic acid control reached 95.79% inhibition. Similarly, in the ABTS assay, the same extracts exhibited 87.94% and 84.146% inhibition for seeds and leaves, respectively, compared to 93.55% for ascorbic acid. Thus, although the extracts demonstrated greater potency in the ABTS assay than in the DPPH assay, the control values remained superior [58].

Additional findings from the evaluation of methanolic and aqueous extracts of *S. molle* leaves and fruits in the DPPH and ABTS assays at 1 mg/mL revealed that the methanolic extracts of the leaves and ripe fruits showed significant radical-scavenging activity (>80%) compared to the control in both assays. Furthermore, in the antioxidant Iron (III) to Iron (II) Reduction Assay, only the methanolic extract of the ripe fruits exhibited a reduction power (1.997 ± 0.001 mmol/g) comparable to that of the control (2.315 ± 0.001 mmol/g) [43].

**Table 1:** Major secondary metabolites present in active extracts or fractions of *S. molle*.

Part of the Plant/ Extract or Fraction	Compound	Concentration	Country of the Sample	Reference
Fruits/Methanolic extract	Masazino-flavanone	1177.65 μg/g	Tunisia	[40]
Fruits/Monosaccharide fraction	Arabinose	40.55%		[41]
	Galacturonic acid	41.15%	Tunisia	
	Fucose	10.90%		
	Galactose	7.40%		
Fruits/Aqueous extract	Chlorogenic acid	0.19 ± 0.01 (mg/gdw)		[42]
	Ellagic acid	0.124 ± 0.002 (mg/gdw)	Peru	
	Quercetin derivatives	0.42 ± 0.06 (mg/gdw)		
Fruits/Methanolic extract		11507.21 ± 90.5 (mg/100 g)	Brazil	[55]
	Fructose	9528.74 ± 46.67 (mg/100 g)	India	
		11829.82 ± 23.73 (mg/100 g)	Sri Lanka	
		9816.07 ± 36.51 (mg/100 g)	Brazil	
	Glucose	6181.37 ± 315.61 (mg/100 g)	India	
		9758.15 ± 330.28 (mg/100 g)	Sri Lanka	
		134.60 ± 3.20 (mg/100 g)	Brazil	
	Piperine	101.10 ± 2.84 (mg/100 g)	India	
		120.67 ± 1.91 (mg/100 g)	Sri Lanka	
		526.72 ± 6.06 (mg/100 g)	Brazil	
	Gallic Acid	657.59 ± 5.25 (mg/100 g)	India	
		168.15 ± 1.43 (mg/100 g)	Sri Lanka	
		144.85 ± 0.71 (mg/100 g)	Brazil	
	Protocatechuic Acid	237.52 ± 0.64 (mg/100 g)	India	
		29.47 ± 0.18 (mg/100 g)	Sri Lanka	
		85.91 ± 2.88 (mg/100 g)	Brazil	
	Epicatechin	89.24 ± 2.04 (mg/100 g)	India	
		38.26 ± 1.28 (mg/100 g)	Sri Lanka	
		115.92 ± 5.00(mg/100 g)	Brazil	
	*p*-Coumaric Acid	151.33 ± 7.07(mg/100 g)	India	
		48.24 ± 1.28(mg/100 g)	Sri Lanka	
Leaves/Essential oils	α-Phellandrene	25.9%	Portugal	[56]
	Limonene	11.7%		
	Myrcene	11.1%		
	β-Phellandrene	10.5%		
	Elemol	9.0%		
Fruits/Essential oils	β-myrcene	51.3%	Portugal	[56]
	Limonene	14.1%		
	α-Phellandrene	14.0%		
	β-Phellandrene	11.0%		
Wood branches/Essential oils	α-Elemol	14.79%	Egypt	[57]
	β-Pinene	13.39%		
	Myrcene	12.26%		
	α-Phellandrene	10.41%		
	Caryophyllene	7.69%		
Seeds/Dichloromethane extract and fractions	Isomasticadienonic acid	n/i	South Africa	[49]
	Masticatrienonate	n/i		
Fruits/Triterpens and biflavanone	3-*epi*-Isomasticadienolalic acid	n/i	Spain	[51]
	Isomasticadienonalic acid	n/i		
	Chamaejasmin	n/i		
Seeds/Methanolic extract	Bis (2-ethylhexyl) phthalate	59.11%	Saudi Arabia	[58]
	n-Hexadecanoic acid	10.84%		
Leaves/Methanolic extract	Squalene	16.87%	Saudi Arabia	[58]
	Azulene	14.88%		
	Lupeol	12.4%		

n/i = no information; mg/gdw = milligrams per gram of dry weight.

### 3.4. Toxicity of Pirul

Due to the traditional uses of *S. molle* as a food condiment and insect repellent, several studies have investigated its safety through toxicity evaluations.

The in vivo assessment of acute dermal exposure to the ethanolic and hexanic extracts from the leaves of *S. molle* var. *areira* at a single dose of 2000 mg/kg of body weight revealed slight signs of erythema and edema on the shaved skin of rats, which disappeared after 48 h. Although no histopathological alterations were observed in internal organs after 14 days, the ethanolic extract caused an increase in locomotor activity in the open field test on day 14. The hexanic extract produced an increase in rearing and arousal behavior, which was reversed after 14 days. Therefore, since both ethanolic and hexanic extracts produced only slight and reversible skin irritation and a transient stimulatory effect in rats, their topical use can be considered safe [59].

The aqueous extract from the leaves of *S. molle*, containing dimeric proanthocyanidins, phenylpropanoid acids, flavan-3-ols, simple organic acids (C6-C1), rutin, and O-glycosylated megastigmane, was evaluated in in vivo acute oral toxicity tests using doses of 5, 50, 300, and 2000 mg/kg. No acute toxic effects were observed in rats. Additionally, no genotoxicity effects were detected in the comet assay or micronucleus tests [60].

Ethanolic extracts from leaves and fruits of *S. molle* var. *areira* were added to the diet of mice at a dose of 1 g/kg body weight/day for 90 days, in order to evaluate the subchronic effect. The extract from fruits produced an increase in the neutrophil count, a decrease in the lymphocyte count, and a reduction in total cholesterol levels, while the extract from the leaves caused an increase in the number of rearings in the open field test. Histopathological examinations revealed no alterations in internal organs. Therefore, the extracts of leaves and fruits of *S. molle* var. *areira* can be considered safe under these conditions [61].

The in vivo evaluation of the toxicity of the ethanolic and hexanic extracts from the fruits and leaves of *S. molle* showed that these extracts could be considered as relatively safe. The extracts were evaluated in rats at doses of 2 g/kg body weight/day for 1 day for acute toxicity and at 1 g/kg body weight/day for 14 days for subacute toxicity. After the subacute period, a significant increase in motor activity was observed in the open field test; however, these changes disappeared after 7 days. Histopathological analyses showed no alterations in the brain, liver, kidney, lung, heart, stomach or intestines of rats at the end of the acute or subacute exposure periods [62].

On the other hand, the fruits of *S. molle*, commonly known as *pink pepper*, have been used as food condiment; however, some toxic and allergic reactions following ingestion or contact have been reported. In the study by Mügge and Morlock et al. [63], moronic acid was identified as one of the main constituents of *S. molle* fruits. This compound has previously been reported to exhibit cytotoxic activity [64]. Nevertheless, factors such as the preparation of the fruits of *S. molle* for use as a condiment, the ingested amount, and the specific cytotoxic effect during oral exposure are crucial considerations in assessing toxicity, and these aspects have not yet been fully clarified [63].

## 4. Discussion

DM is a chronic metabolic disease that has been recognized as a global epidemic, characterized by prolonged hyperglycemia that leads to several severe lengthy health complications [65]. Despite the availability of approved drugs for its treatment, there is still no cure for DM. Therefore, research into natural products has become particularly relevant for the discovery of novel and effective drugs due to their multicomponent, multitargeted, and fewer side effects, showing that these could be useful in the treatment of DM [66]. One of the various antidiabetic mechanisms that has gained attention in recent years is the inhibition of enzymes α-amylase and α-glucosidase to manage the blood glucose levels. Both α-glucosidase and α-amylase enzyme inhibitors can suppress peaks of postprandial glucose. α-glucosidase breaks down starch and disaccharides, while α-amylase breaks internal α-1, 4-glycosidic linkages of starch into glucose and maltose. Thus, the antidiabetic effect of these enzymes lies in delaying glucose absorption through inhibition of these enzymes in the digestive organs, mainly in the small intestine [67]. In the present review, a dose-dependent inhibitory activity on both enzymes was highlighted for the methanolic extract of *S. molle* fruits originating from Tunisia. However, different results were observed in extracts from other parts of the plant and from samples collected in other countries, where inhibition on the α-glucosidase enzyme was generally more potent than that of α-amylase (Table 1). These differences may relate to the chemical composition of the samples. In particular, the Tunisian sample contained Masazino-flavanone as its major compound, whereas this compound was absent in other samples. Therefore, the dual inhibitory enzymatic activity observed may be attributed to the presence of Masazino-flavanone, which is consistent with reports describing the potent inhibitory activity on glucosidase enzymes exhibited by flavanones, where the structural B-ring is determinant [68].

Other mechanisms recognized as key in the pathogenesis of DM and prediabetes include oxidative stress and inflammation. Both are physiological processes with protective functions. Oxidative stress through reactive oxygen species (ROS) participates in the removal of pathogens and the signals of tissue repair. Inflammation reacts to injury or infection by isolation and elimination of the damage caused and also initiates the healing process [69]. However, an imbalance between free radical production and the antioxidant system leads to a reduction in peripheral insulin sensitivity and favors the development of DM through multiple molecular pathways, altering functional and structural molecules, preceding tissue injury and dysfunction. The inflammation response, initiated by tissue damage, induces the release of cytokines and chemokines that promote intracellular signaling pathways, as kinases and transcription factors. These mechanisms contribute to both macrovascular and microvascular complications associated with poor glycemic control [70,71]. In this context, we reviewed the antioxidant and the anti-inflammatory properties of *S. molle* from samples deriving from different parts of the plant, as well as from samples deriving originally from different countries. Various extracts and fractions of *S. molle* exhibited significant antioxidant and anti-inflammatory activities in both in vitro and in vivo assays, particularly those obtained from fruits and seeds (Table 1). The main compound detected in these active extracts and fractions was Masazino-flavanone from a sample collected in Tunisia [40]. Therefore, the antidiabetic effect exhibited by the methanolic extract of the fruits of *S. molle* with a majority composition of Masazino-flavanone could be considered integral in that they are due to the inhibitory enzymatic properties, as well as to the antioxidant and the anti-inflammatory properties exhibited and reviewed in this present contribution. Furthermore, recent studies have reported cardiopreventive effects of the Masazino-flavanone in in vivo assays [72], which is especially relevant since cardiovascular diseases represent one of the major causes of morbidity and mortality in persons living with DM [73]. Thus, in the present review, the samples of the fruits from Tunisia stand out from the rest for their chemical profile and biological efficacy. Another interesting sample, because of its anti-inflammatory effects, was the sample from South Africa, with a principal content of Isomasticadienonic acid [49]. This compound has been identified as a selective inhibitor of the enzyme 11β-hydroxysteroid dehydrogenase 1, which converts inactive cortisone into active cortisol. This enzymatic inhibition contributes to antidiabetic activity by regulating glucose and fatty acid metabolism [74].

Regarding antioxidant properties, and in addition to the previous comments, in the present contribution, samples of *S. molle* from different countries (Table 1) were reviewed. The main chemicals with antioxidant activities were essential oils, principally comprising α and β-Phellandrene. However, their overall antioxidant activity was relatively low, which is consistent with published reports that mention that monoterpenes, even in high amounts, showed low or almost ineffective activity in some antioxidant assays, such as DPPH and ABTS [75].

Concerning the toxicological effects of pirul, the literature reviewed primarily addresses acute effects resulting from ingestion or contact. Although some reversible skin irritation and alterations in the motor system were observed in the experimental animals, extracts from pirul could be considered safe. Nevertheless, the doses tested are not homogeneous among reports, and the chemical composition of some of them is not defined. Therefore, the toxicological profile of *S. molle* still requires further experimental evidence.

Other species belonging to the same botanical family, Anacardiaceae, have also demonstrated antidiabetic properties. One of them is the species *Pistacia lentiscus*, a species that thrives in the Mediterranean region and has exhibited antioxidant, antiatherogenic, anticancer, and antibacterial properties. In traditional medicine, it has been used in the treatment of DM. Recent studies have revealed its antidiabetic properties through different mechanisms, such as the inhibition of crucial gastrointestinal enzymes involved in carbohydrate digestion and absorption (α-amylase, α-glucosidase); the regulation of glucocorticoid metabolism by inhibiting pancreatic lipase enzymes; hypoglycemic activity by substantially reducing blood glucose levels through oral administration of 50 mg/kg and 125 mg/kg of ethanolic extract of leaves and fruits in in vivo experiments; and hypolipidemic effects in animal models and human subjects. However, the limitation of this species so far is that the compounds responsible for its antidiabetic properties have not yet been identified [76]. Another relevant member of the Anacardiaceae family with antidiabetic properties is mango (*Mangifera indica*). Recent clinical studies indicate that daily mango intake for 4 weeks increased insulin sensitivity and also contributed to the reduction in the amount of insulin required to maintain glucose in people with chronic low-grade inflammation. These effects are likely linked to modifications in cellular redox activities rather than to the inflammatory process [77]. Similar to *Pistacia lentiscus*, the bioactive compounds responsible for these effects remain unidentified. Therefore, when comparing these two species with pirul, all of them belonging to Anacardiaceae, although these species show evidence of antidiabetic properties in vivo, the active compounds have not yet been elucidated. In the case of pirul, additional studies such as those conducted on *Pistacia lentiscus* and mango to evaluate its glucose-lowering potential appear to be a priority.

Finally, it is interesting to note the clear variations in chemical composition that occur in the samples of *S. molle* species that thrive in different countries around the world (Table 1). This observation is also consistent with previous publications [56] that mention that the main chemical components of *S. molle* may differ due to the specific climatic and soil conditions of the region of origin, which could be favoring intraspecific differences. Furthermore, some authors previously proposed the existence of different chemotypes of *S. molle* that must be confirmed by pending studies involving its populations and individuals [78].

Therefore, this review is distinguished by its original selection of the object of study, as the most well-known medicinal uses of the pirul do not relate to diabetes mellitus (DM). Furthermore, the pirul is not considered edible per se, but its accessibility as a resource makes it attractive for use. Additionally, this contribution is distinguished by its analysis of the potential active chemical compounds in DM and their geographical availability, which, to our knowledge, is being reported for the first time.

## 5. Future Directions

Natural products and their derivatives have made remarkable contributions to the field of medicine, offering a wide array of new pharmacological entities for the treatment of diverse diseases. Although several specific medications exist for the management of DM, traditional medicine remains the first-line therapeutic approach in many marginalized and rural communities worldwide, where the use of medicinal plants is especially prevalent. Furthermore, the WHO recognizes traditional medicine not only for its contribution to health but also to the well-being, people-centered health care, and universal health coverage.

The case of *S. molle* is interesting not only for the multitarget experimental properties exhibited so far in the literature reviewed but also for its advantages as a potential raw material for drug development. These advantages include its wide worldwide distribution and easy accessibility in regions where it naturally grows. In the present review, the enzyme inhibition, anti-inflammatory, and antioxidant properties of *S. molle* were identified as potentially useful in the management of DM. Nevertheless, future studies should also address other key therapeutic targets to DM, such as hypoglycemic activity, which, to the best of our knowledge, has not yet been investigated. Research in this direction could provide more precise and conclusive evidence regarding the therapeutic use of pirul in DM. Additionally, studies exploring insulin resistance and gut microbiota modulation represent other research directions that have also been demonstrated to be crucial in the pathogenesis of DM.

Another important yet insufficiently explored aspect concerns the toxicological profile of *S. molle*. Although some preliminary toxicological studies have been conducted, as discussed in this review, neither the active nor the potentially toxic compounds have been fully characterized, and therapeutic dosage ranges remain undefined. To fill these gaps, exhaustive identification of active compounds should be performed using advanced spectroscopic and spectrometric techniques necessary for their complete identification, as well as crystallography studies when applicable. Recent studies demonstrated that HPLC-DAD-ESI-QTOF-MS analysis would be very effective for the determination of phenolics and other polar compounds. Isomasticadienonic acid and Masazino-flavanone are two compounds indicated in the present review as related to the antidiabetic properties of pirul, but the identification of active compounds in the rest of the fractions or polar extracts is still pending. Furthermore, future research should focus on the standardization of therapeutic doses of *S. molle* and performance of in vivo studies to validate efficacy. A complete pharmacokinetic profile that evaluates the safety, tolerability, and efficacy of pirul will be essential to support potential clinical studies and future therapeutic applications.

As can be seen so far, one of the most relevant contributions of this review is precisely to draw attention to the potential therapeutic effects of pirul for the treatment of DM. To date, experimental evidence is still limited, making *S. molle* a strong candidate for more rigorous research in the field of natural product-based drug discovery.

## 6. Conclusions

*S. molle* has exhibited properties as an enzyme inhibitor of α-glucosidase and α-amylase, as well as an anti-inflammatory and an antioxidant, which allow it to be considered as a future alternative in the treatment of DM, with the advantage of possessing multitarget antidiabetic actions. However, this promising profile is tempered by significant limitations in the scientific record. Critically, negative results have also been reported, with some studies finding that specific aqueous and methanolic extracts showed no activity in certain in vitro models. This underscores that the bioactivity is highly dependent on factors such as the plant’s geographical origin, the specific plant part used, the extraction solvent, and the biological assay employed. Nonetheless, particularly promising is the identification of specific compounds, such as Isomasticadienonic acid, with a defined mechanism of action (e.g., 11β-HSD1 inhibition), alongside robust in vitro evidence for α-glucosidase inhibition. Another auspicious active compound is Masazino-flavanone, which, in addition to the in vivo and in vitro antioxidant and anti-inflammatory properties, has cardiopreventive properties by reducing the high-cardiac-risk parameters of myocardial infarction. Therefore, to date, experimental evidence places *S. molle* as a potential candidate to be part of the DM arsenal in the future. An immediate and rigorous toxicological profile and clinical trials in persons with DM are still pending.

## Data Availability

No new data were created or analyzed in this study. Data sharing is not applicable to this article.
